# Virus-induced plasma membrane aquaporin *PsPIP2;1* silencing inhibits plant water transport of *Pisum sativum*

**DOI:** 10.1186/s40529-016-0135-9

**Published:** 2016-08-06

**Authors:** Juanjuan Song, Guoliang Ye, Zhengjiang Qian, Qing Ye

**Affiliations:** 1 Key Laboratory of Vegetation Restoration and Management of Degraded Ecosystems, South China Botanical Garden, Chinese Academy of Sciences, 723 Xingke Road, Tianhe District, Guangzhou, 510650 China; 2 Guangdong Provincial Key Laboratory of Applied Botany, South China Botanical Garden, Chinese Academy of Sciences, 723 Xingke Road, Tianhe District, Guangzhou, 510650 Guangdong China; 3 University of Chinese Academy of Sciences, 19A Yuquan Road, Beijing, 100049 China

**Keywords:** Cell pressure probe, Hydraulic conductivity, Plant water relations, VIGS, Water channels

## Abstract

**Background:**

Aquaporins (AQPs) are known to facilitate water transport across cell membranes, but the role of a single AQP in regulating plant water transport, particularly in plants other than *Arabidopsis* remains largely unexplored. In the present study, a virus-induced gene silencing (VIGS) technique was employed to suppress the expression of a specific plasma membrane aquaporin *PsPIP2;1* of Pea plants (*Pisum sativum*), and subsequent effects of the gene suppression on root hydraulic conductivity (Lp_r_), leaf hydraulic conductivity (*K*_*leaf*_), root cell hydraulic conductivity (Lp_rc_), and leaf cell hydraulic conductivity (Lp_lc_) were investigated, using hydroponically grown Pea plants.

**Results:**

Compared with control plants, VIGS-PsPIP2;1 plants displayed a significant suppression of *PsPIP2;1* in both roots and leaves, while the expression of other four PIP isoforms (*PsPIP1;1*, *PsPIP1;2*, *PsPIP2;2*, and *PsPIP2;3*) that were simultaneously monitored were not altered. As a consequence, significant declines in water transport of VIGS-PsPIP2;1 plants were observed at both organ and cell levels, i.e., as compared to control plants, Lp_r_ and *K*_*leaf*_ were reduced by 29 %, and Lp_rc_ and Lp_lc_ were reduced by 20 and 29 %, respectively.

**Conclusion:**

Our results demonstrate that *PsPIP2;1* alone contributes substantially to root and leaf water transport in Pea plants, and highlight VIGS a useful tool for investigating the role of a single AQP in regulating plant water transport.

**Electronic supplementary material:**

The online version of this article (doi:10.1186/s40529-016-0135-9) contains supplementary material, which is available to authorized users.

## Background

Plant water relations are continually challenged by diverse environmental stimuli, such as light, temperature, soil water availability, and atmospheric humidity. To keep water homeostasis, plants need to respond promptly to the ever-changing environments via regulating water transport at cellular, tissue, organ, and whole plant level (Aroca et al. 2012; Bramley et al. 2009; Chaumont and Tyerman 2014; Chevalier and Chaumont 2015; Henry et al. 2012; Luu and Maurel 2005). Aquaporins (AQPs) are trans-membrane proteins that facilitate rapid and passive water transport across cell membranes. According to sequence homology and sub-cellular localizations, plants AQPs can be classified into seven subfamilies, i.e., plasma membrane intrinsic proteins (PIPs), tonoplast intrinsic protein (TIP), NOD26-like intrinsic proteins (NIPs), small intrinsic proteins (SIPs), X-intrinsic proteins (XIPs), GlypF-like intrinsic proteins (GIPs), and hybrid intrinsic proteins (HIPs) (Anderberg et al. 2011; Gustavsson et al. 2005; Johanson et al. 2001; Li et al. 2014). Among them, PIPs constitute the largest number and can be further divided into two subgroups named PIP1 and PIP2 (Ayadi et al. 2011; Chaumont et al. 2000; Johansson et al. 2000).

The role of AQPs in regulating plant water transport has been abundantly documented, and PIPs represent the most likely candidates for protein-mediated hydraulic conductivity in plants (Heinen et al. 2009; Maurel et al. 2008). The contribution of AQPs to plant hydraulic conductivity has been tested by variable approaches. The first notion that AQPs involving in plant water transport was raised from experiments showing that root water transport can be substantially inhibited by AQP blocker, i.e., mercurial regents (Javot and Maurel 2002; Maggio and Joly 1995; Zhang and Tyerman 1999). Because mercury compound showed inhibitive effects in general on other physiological processes besides blocking AQPs, more specific approaches involved the use of transgenic plants with altered expression of targeted PIPs were employed (Jang et al. 2007; Javot et al. 2003; Lee et al. 2012; Postaire et al. 2010; Secchi and Zwieniecki 2014; Yu et al. 2005). For instance, over-expression of *Arabidopsis PIP1b* in tobacco improved plant vigor under favorable growth condition (Aharon et al. 2003). Low temperature induced reductions in cell hydraulic conductivity was alleviated by over-expressing *AtPIP2;5* in *Arabidopsis* plants (Lee et al. 2012). In grapevine, it was found that the over-expression of a root specific AQP *VvPIP2;4N* enhanced water transport at the whole plant level (Perrone et al. 2012). By contrast, hydraulic conductivity of root cortex cell was reduced by 25–30 % in *PIP2;2* knockout mutant of *Arabidopsis* plants (Javot et al. 2003), and a reduction of about 20 % in the relative water flux into rosette leaves was found in these mutants (Da Ines et al. 2010). Similarly, disruption of *AtPIP1;2* resulted in a significant decrease (by 20–30 %) in root hydraulic conductivity of *Arabidopsis* (Postaire et al. 2010), while PIP1 and PIP2 double antisense *Arabidopsis* plants had a threefold decrease in the root hydraulic conductivity (Martre et al. 2002). All these pioneer findings pointed to the important roles of AQPs in regulating water transport across diverse species, while the contribution of a single AQP to hydraulic conductions in plants other than *Arabidopsis* remains to be explored.

Virus-induced gene silencing (VIGS) is a reverse genetics technology that can produce a rapid, sequence-specific knockdown phenotype for the target gene (Burch-Smith et al. 2004). To this end, a fragment of the target gene is inserted into a viral delivery vector which is used to infect plants. During the inoculation, virus replication triggers the natural defense mechanisms of plants to suppressing virus replication, which is also result in specific degradation of mRNAs from the endogenous gene that is targeted for silencing (Baulcombe 1999; Lu et al. 2003). Therefore, compared with other transgenic methods, VIGS technology represents a simple but attractive reverse-genetics tool for gene functional studies (Pflieger et al. 2013). In addition, VIGS does not need to develop stable transformants, thus can be used to study the function of genes that might be fatal for plants when such functions are impaired in stable transformed lines (Burch-Smith et al. 2004; Purkayastha and Dasgupta 2009). With these advantages, VIGS technology has been broadly applied for functional studies of specific genes across a number of plant species including Tobacco, *Arabidopsis*, Tomato, Rice, and Pea plants (Constantin et al. 2004; Fragkostefanakis et al. 2014; Purkayastha et al. 2010; Senthil-Kumar and Mysore 2014).

## Results and discussion

**Fig. 1:**
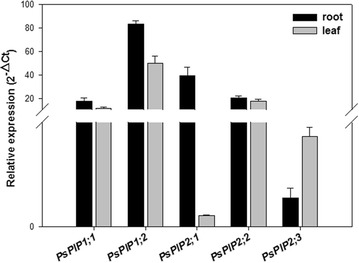
Expression profiles of the five cloned *PsPIPs* in roots and leaves of Pea (*Pisum sativum*) plants

In this study, five PIP isoforms (*PsPIP1;1*, *PsPIP1;2*, *PsPIP2;1*, *PsPIP2;2*, and *PsPIP2;3*) were identified and cloned in *Pisum sativum*. In the preliminary trials, our quantitative real-time PCR (q-RT-PCR) results revealed that the expression of *PsPIP2;1* was the highest in roots among the three study PIP2s (Fig. 1). Along with previous findings that *PsPIP2;1* showed marked water transport activity when expressed in *Xenopus* oocytes (Schuurmans et al. 2003), and its expression pattern displayed a tight correlation with the diurnal change in root hydraulic conductivity (Beaudette et al. 2007), we therefore chose *PsPIP2;1* as the primarily target gene to explore its contribution to water transport in Pea plants. Firstly, we employed the VIGS method to suppress the expression of *PsPIP2;1*, which was quantified using q-RT-PCR, with the expression of the other four PIPs being monitored simultaneously. Subsequently, changes in root and leaf hydraulic conductivities of VIGS-PsPIP2;1 plants at both organ and cell levels were determined using pressure chamber and cell pressure probing techniques, respectively, and the role of *PsPIP2;1* in regulating Pea plant water transport was discussed.

**Fig. 2:**
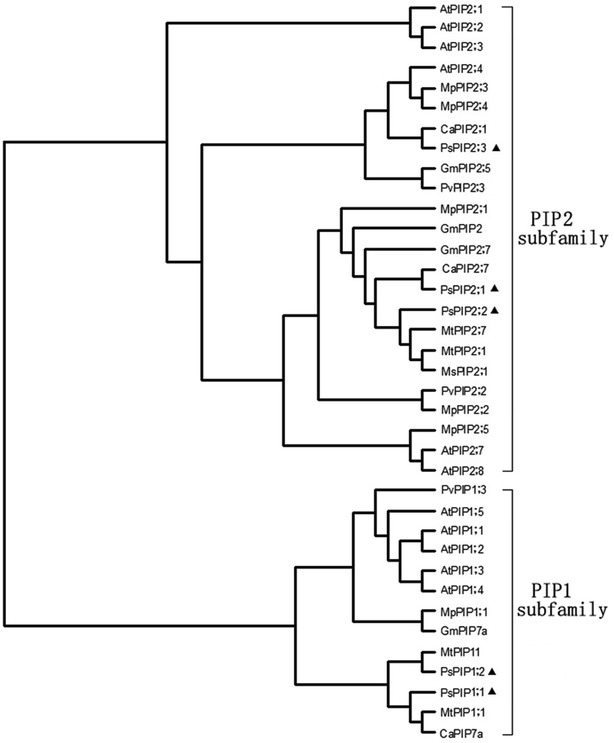
Phylogenetic tree of AQPs from *Pisum sativum* sequences and other plants generated in MEG5.1 software. Subfamilies are labeled by* brackets* at the* right side*. Sequences of *P. sativum* are indicated by *filled triangles*. Information of the known 32 AQP sequences and GenBank accession numbers used are as follows: *Arabidopsis thaliana*, *AtPIP1;1* (AEE80201), *AtPIP1;2* (AEC10622), *AtPIP1;3* (AEE27312), *AtPIP1;4* (AEE81879), *AtPIP1;5* (AEE84748), *AtPIP2;1* (AEE79084), *AtPIP2;2* (AEC09362), *AtPIP2;3* (AEC09363), *AtPIP2;4* (AED97364), *AtPIP2;7* (AEE86464), *AtPIP2;8* (AEC06543); *Mimosa pudica*, *MpPIP1;1* (BAD90696), *MpPIP2;1* (BAD90697), *MpPIP2;2* (BAD90698), *MpPIP2;3* (BAD90699), *MpPIP2;4* (BAD90700), *MpPIP2;5* (BAD90701); *Cicer arietinum*, *CaPIP7a*(XM_004490904), *CaPIP2;1*(XM_004496224), *CaPIP2;7*(XM_004505936); *Glycine max*, *GmPIP2;7*(XM_003538126), *GmPIP2* (XM_003540128), *GmPIP;7a* (XM_003544062), *GmPIP2;5* (XM_003556184); *Medicago truncatula*, *MtPIP11*(XM_003600815), *MtPIP1;1*(AF386739), *MtPIP2;1*(AY059380), *MtPIP2;7*(XM_003606335); *Phaseolus vulgaris*, *PvPIP1;3* (DQ855475), *PvPIP2;2* (EF624001), *PvPIP2;3* (EF624002); *Medicago sativa* subsp. Falcate, *MsPIP2;1*(FJ607305); *Pisum sativum*, *PsPIP1;1* (X54357), *PsPIP1;2* (KF770828), *PsPIP2;1* (AJ243307), *PsPIP2;2* (KF770829), *PsPIP2;3* (KF770830)

**Fig. 3:**
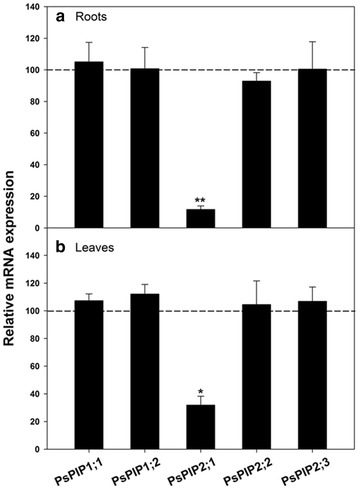
Relative expression of *PsPIP* genes in roots (*a*) and leaves (*b*) of control plants and VIGS-PsPIP2;1 plants determined by quantitative real-time PCR. Values are mean ± SD of three independent replicates. *Asterisks* or *two asterisks* (* or **) represent a significant difference between VIGS-PsPIP2;1 plants and the control plants at P < 0.05 or P < 0.01

There is increasing evidence supporting the crucial roles of AQPs in regulating plant water transport (Aroca et al. 2012; Chevalier and Chaumont 2015; Li et al. 2014). However, due to high multiplicity of plant AQP isoforms, the contribution of a specific AQP gene to hydraulic conductivity of non- *Arabidopsis* plants remains largely unknown. In the present study, five full-length cDNAs of plasma membrane aquaporins (PIPs) were isolated from Pea plants. Among them, two genes belong to the PIP1 subfamily, and the other three belong to the PIP2 subfamily, which were designated as *PsPIP1;1, PsPIP1;2, PsPIP2;1, PsPIP2;2* and *PsPIP2;3*, respectively (Fig. 2). Taking the advantage of VIGS method, we intended to investigate the contribution of *PsPIP2;1* to water transport in both roots and leaves of Pea plants. Because AQPs constitute a large and highly divergent protein family in plants, it is important to carefully analyze possible compensation effects by closely related isoforms when studying the function of a specific AQP through modifying its expression (Heinen et al. 2009). For example, the transcript levels of endogenous PIPs was noticeably affected by the over-expression of *PIP1;4* and *PIP2;5* in *Arabidopsis* plants under water stress conditions, it is therefore difficult to attribute the observed phenotypes to the abundance change of target gene or to the altered expression of other endogenous AQPs (Jang et al. 2007). In the present study, our q-RT-RCR analysis confirmed that the mRNA expression of *PsPIP2;1* was significantly inhibited in both roots and leaves without altering the expression of the other four PIP isoforms (Fig. 3). Therefore, it might be reasonable to attribute changes in plant hydraulic conductivity to the suppression of *PsPIP2;1*, although alterations in the expression of other PIPs that are similar to *PsPIP2;1* but not yet identified cannot be completely ruled out.

**Fig. 4:**
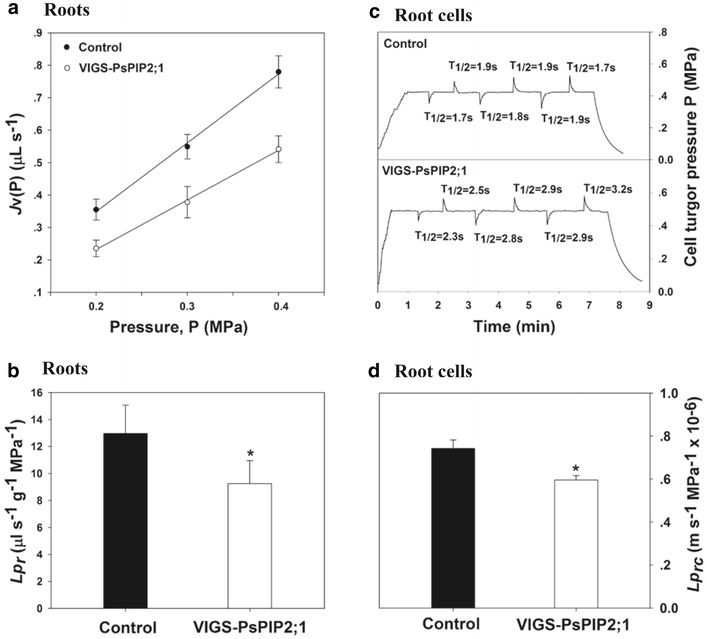
Virus induced *PsPIP2;1* silencing led to reductions in root hydraulic conductivities. **a** Representative pressure-to-flow relationship measured in roots of control plants and VIGS-PsPIP2;1 plants. *J*_*v*_ represents the rate of exuded sap flow through roots. **b** Root hydraulic conductivity at organ level. **c** Typical hydrostatic relaxation curves as measured by a cell pressure probe on root cells. **d** Root hydraulic conductivity at cell level. Values are mean ± SD (n = 6–9 plants or 30–60 cells). *Asterisks* (*) represent a significant difference between control plants and VIGS-PsPIP2;1 plants at P < 0.05

At the root level, a reduction of Lp_r_ by 29 % was observed in VIGS-PsPIP2;1 plants compared with control plants (Fig. 4b). Cell volume (V), surface area (A), turgor pressure (P), and elasticity (ε) were not affected significantly by the virus-induced *PsPIP2;1* silencing (Table 1), but the half-time of water exchange (T_1/2_) increased from 1.8 s (on average) to 2.6 s (Fig. 4c), resulting in a decrease of Lp_rc_ by 20 % in VIGS-PsPIP2;1 plants compared with the control plants (Fig. 4d). Our results are comparable to observations in *Arabidopsis* knockout mutants. On one hand, both the cell and the root hydraulic conductivity of Pea plants measured in this study were at the same magnitude as those of *Arabidopsis* plants (Javot et al. 2003; Postaire et al. 2010); on the other hand, a reduction of 20–30 % in root hydraulic conductivity was observed in *PIP1;2* mutants (Postaire et al. 2010), and hydraulic conductivity of root cortex cell was reduced by 25–30 % in *PIP2;2* mutant (Javot et al. 2003). These findings pointed to a substantial contribution of a single AQP isoform to root water transport. Along with the finding that the expression of *PsPIP2;1* was positively correlated with the diurnal changes in root hydraulic conductivity of Pea plants (Beaudette et al. 2007), here our results provided further evidence that *PsPIP2;1* was indeed involved in mediating root water transport in Pea plants.

**Table 1:** Cell pressure probe measurements of root cortex cells and leaf epidermal cells of control plants and virus induced *PsPIP2;1* silencing plants (VIGS-PsPIP2;1)

	Control	VIGS-PsPIP2;1
Root cortical cell		
Turgor pressure, P (MPa)	0.38 ± 0.08 a	0.37 ± 0.06 a
Cell volume, V (m^3^)	1.7 ± 0.3E−13 a	1.6 ± 0.2E−13 a
Cell surface area, A (m^2^)	2.2 ± 0.4E−08 a	2.1 ± 0.3E−08 a
ε (MPa)	4.0 ± 1.0 a	3.9 ± 1.2 a
T_1/2_ (s)	1.8 ± 0.1 a	2.6 ± 0.6 b
Leaf epidermal cell		
Turgor pressure, P (MPa)	0.34 ± 0.05 a	0.32 ± 0.08 a
Cell volume, V (m^3^)	1.1 ± 0.2E−13 a	1.2 ± 0.1E−13 a
Cell surface area, A (m^2^)	1.7 ± 0.5E−08 a	1.6 ± 0.4E−08 a
ε (MPa)	2.2 ± 1.1 a	2.3 ± 1.2 a
T_1/2_ (s)	1.2 ± 0.2 a	1.7 ± 0.4 b

Results are presented as mean ± SD (n = 30–60 cells). Different letters indicate significant differences (P < 0.05)

**Fig. 5:**
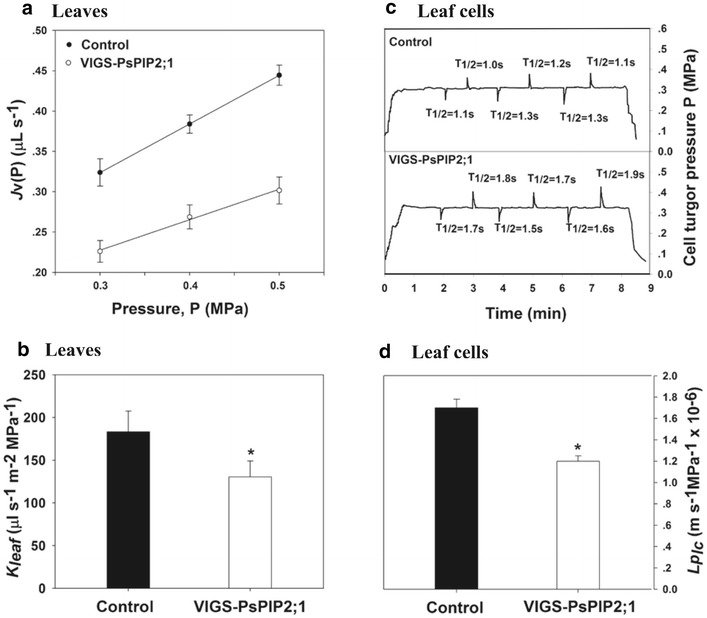
Virus induced *PsPIP2;1* silencing led to reductions in leaf hydraulic conductivities. **a** Representative pressure-to-flow relationship measured in leaves of control plants and VIGS-PsPIP2;1 plants. *J*_*v*_ represents the rate of exuded sap flow through leaves. **b** Leaf hydraulic conductivity at organ level. **c** Typical hydrostatic relaxation curves as measured by a cell pressure probe on leaf cells. **d** Leaf hydraulic conductivity at the cell level. Values are mean ± SD (n = 6–9 plants or 30–60 cells). *Asterisks* (*) represent a significant difference between control plants and VIGS-PsPIP2;1 plants at P < 0.05

The values of leaf hydraulic conductivities at both organ and cell levels that we measured in Pea plants were comparable to those of many other species such as Maize (Kim and Steudle 2007), *Arabidopsis* (Prado et al. 2013), and Cucumber (Qian et al. 2015). As found in roots, significant reductions in leaf hydraulic conductivities were observed when the expression of *PsPIP2;1* was suppressed. For instance, *K*_*leaf*_ were 183.3 ± 24.1 and 130.4 ± 18.8 μL s^−1^ m^−2^ MPa^−1^ in the control and VIGS-PsPIP2;1 plants, respectively, indicating that *K*_*leaf*_ decreased by 29 % (Fig. 5b). Without significant differences in cell geometry, turgor pressure, and cell wall elasticity between the control and VIGS-PsPIP2;1 plants (Table 1), we found that T_1/2_ increased (on average) from 1.2 to 1.7 s (Fig. 5c), indicating a decrease of leaf cell hydraulic conductivity by 29 % in VIGS-PsPIP2;1 plants compared with the control plants (Fig. 5d). In leaves, it has been shown that alteration of AQP expression significantly affected leaf hydraulic conductivity, indicating the crucial roles of AQPs in plant leaf water transport (Cochard et al. 2007; Ding et al. 2004; Lopez-Berenguer et al. 2008; Muries et al. 2013). In the present study, we found the silencing of the *PsPIP2;1* resulted in a reduction of 29 % the leaf hydraulic conductivities at both organ and cell levels, which was consistent with findings of previous studies. For instance, employing a deuterium tracer method, Da Ines et al. (2010) demonstrated that water flux into the *Arabidopsis* rosette was significantly reduced by about 20 % in*PIP2;1* and *PIP2;2* knockout plants. By measuring *Arabidopsis* rosette water flux of three single PIP (*PIP1;2*, *PIP2;1*, or *PIP2;6*) knockout mutants, Prado et al. (2013) observed a significant reduction of water flux by 16–35 % as compared to wild type, and the authors concluded that *PIP1;2*, *PIP2;1*, and *PIP2;6* are important contributors of AQP-mediated rosette water transport. In this study, we noted that the expression of *PsPIP2;1* in leaves was relatively low, still the reduction in leaf hydraulic conductivity of VIGS-PIP2;1 plants was significant. Therefore, except for expression level, the activity and/or localization of *PsPIP2;1* might be critical in regulating Pea plant leaf water transport, as being pointed out by different researchers in a number of recent studies (Chevalier and Chaumont 2015; Kaneko et al. 2015), which deserve further investigations.

## Conclusions

Our results demonstrated that the expression of *PsPIP2;1* in Pea plants was specifically suppressed through the VIGS method. As a result, both root and leaf hydraulic conductivities were significantly reduced in *PsPIP2;1*-silenced plants compared with control plants. Consistent with previous findings that *PsPIP2;1* showed marked water transport activity when expressed in *Xenopus oocytes*, and displayed a tight correlation with the diurnal change in root hydraulic conductivity, our results provided further evidence that *PsPIP2;1* play an important role in regulating Pea plant water transport. However, precise mechanisms by which this AQP mediates plant water transport remain to be explored. For instance, whether *PsPIP2;1* had a tissue specific expression pattern in root endodermis and/or leaf bundle sheath that are proven to be critical in the pathway of plant water transport, as well as the responsiveness of *PsPIP2;1* to abiotic stresses (e.g., drought stress), all merit future investigations.

## Methods

### Plant material and growth conditions

Pea plants (*P. sativum* L. line *JI992*) used in this study was obtained from National Key Laboratory of Plant Molecular Genetics, Institute of Plant Physiology and Ecology, Chinese Academy of Sciences, Shanghai, China. Seeds were germinated in wet filter paper in covered Petri dishes for 3 days at room temperature in the dark. Then seedlings were transferred to a hydroponic culture plastic box (7 L) filled with modified Hoagland solution (pH = 6.0; 1.25 mM Ca(NO_3_)_2_, 1.25 mM KNO_3_, 0.5 mM MgSO_4_, 0.25 mM KH_2_PO_4_; micronutrients: 10 µM H_3_BO_3_, 1 µM MnSO_4_, 0.5 µM ZnSO_4_, 0.05 µM (NH_4_)_6_Mo_7_O_24_ and 0.4 µM CuSO_4_) following Jelali et al. (2010). The nutrient solution was aerated with the aid of aquarium diffusers. One week later, seedlings were transferred to 37 L boxes (15 plants per box) filled with the same nutrient solution that was replaced weekly. Growing conditions in the growth chamber were 16 h light/8 h dark photoperiod, 18/20 °C, 65 % humidity, and a photon flux density of 200–300 µM m^−2^ s^−1^. Plants used in the experiments were six- to seven-week old.

### RNA extraction and *PsPIP* genes identification

Total RNA was extracted from roots and leaves of Pea plants using Trizol regent (Invitrogen, Grand Island, NY, USA) following the manufacturer’s instructions. The concentration of RNA was quantified by spectrophotometrical measurement at λ = 260 nm, and its integrity was checked on agarose gels. First strand cDNA was synthesized from 2 µg of total RNA using GoScript reverse transcription regent Kit (Promega, Madison, WI, USA).The synthesized cDNA was amplified by polymerase chain reaction (PCR) using oligo(dT) and degenerate oligonucleotide primers (Additional file 1: Table S1) which were designed from the known sequences of different plant PIP genes. The PCR products were gel-purified and sub-cloned into pMD18-T vector (Takara, TAKARA Biotechnology Co. Ltd, Dalian, China), and the constructed plasmids were transformed into *E. coli* DH5α. The positive clones were sequenced and analyzed. Next, 5′-rapid amplification of cDNA ends (RACE) was applied to clone the 5′-end sequences of the PIP genes. Sequences analyses with database were performed at NCBI (http://www.ncbi.nlm.nih.gov/) using the BLAST network services, and a phylogenetic tree was generated in MEG5.1 software (http://www.megasoftware.net) to test the evolutionary relationships.

### The Silencing of *PsPIP2;1* in Pea plants

To optimize the VIGS method, Constantin et al. (2004) transferred the RNA1 and RNA2 expression cassettes of a *Pea early browning virus* (PEBV) to the binary agrobacterium vector pCAMBIA1300. Then, pCAMBIA1300-derived plasmid with the expression cassette of RNA1 was named as pCAPE1, and pCAMBIA1300-derived plasmid with the expression cassette of RNA2-GFP was named as pCAPE2-GFP. In the present study, sequence of GFP in pCAPE2-GFP was replaced with cDNA fragment of *P. sativum* phytoene desaturase (PDS) gene and with partial encoding region of *PsPIP2;1* plus 3′UTR sequence to obtain pCAPE2-PDS and pCAPE2-PsPIP2;1, respectively. Also, a vector control plasmid, pCAPE2-Con was constructed by replacing the GFP sequence of pCAPE2-GFP with a fragment derived from the cDNA of Bean yellow mosaic virus (AJ622899). Next, the constructed plasmids including pCAPE1, pCAPE2-PDS, pCAPE2-PsPIP2;1, and pCAPE2-Con were transformed separately into *Agrobacterium tumefaciens* GV3101 using the freeze–thaw method (Hofgen and Willmitzer 1988). Two-week old Pea plants were infiltrated at the abaxial side of the youngest pair of leaves with agrobacterium cultures carrying pCAPE1 and the pCAPE2-derived plasmids at a 1:1 ratio. Plants were separated into three groups that were subsequently inoculated with three different agrobacterium cultures: (1) pCAPE2-PDS, which served as an indicator of gene silencing, in that PDS silenced plants had photo-bleached leaves (as a result of lacking carotenoids and destruction of chlorophyll by photo-oxidation), and this phenotype was associated with a significant reduction in *PsPDS* mRNA (Kumagai et al. 1995); (2) pCAPE2-PsPIP2;1 to silence the target *PsPIP2;1* gene; and (3) pCAPE2-Con as the control. When the target gene was silenced, as indicated by the photo-bleached leaves of PDS silenced plants, the shoots of *PsPIP2;1* silenced plants were labeled at the position where the photo-bleached phenotype began to appear (Additional file 2: Figure S1). Meanwhile, roots of *PsPIP2;1* silenced plants were cut back to approximately 3 cm and root growth was allowed to re-initiate. Then plants were grown in the growth chamber for additional 2–3 weeks to allow the production of newly emerged leaves and regenerative roots, which were used in subsequent experiments.

### Quantitative real-time PCR (q-RT-PCR) analyses

Total RNA extraction, concentration and integrity were determined as described above. First strand cDNA was synthesized using primeScript RT regent Kit (TakaRa, TAKARA Biotechnology Co. Ltd, Dalian, China) following manufacturer’s instructions, including a special step for genomic DNA elimination. Quantitative PCR analysis was conducted on an ABI 7500 Real-Time system using a SYBR Green *Premix Ex*-*Taq*™II Kit (TakaRa, TAKARA Biotechnology Co. Ltd, Dalian, China) with *PsPIP* gene specific primers (Additional file 3: Table S2). The reaction mixture had a final volume of 20 µL, containing 10 µL 2× SYBR *Premix Ex Taq*™II, 0.4 µM of each primer, 0.4 µL 50× ROX Reference Dye II and 2 µL of tenfold dilution cDNA. The PCR conditions were: 30 s at 95 °C for pre-denaturation; 40 cycles of 5 s at 95 °C, 34 s at 60 °C. The melt-curve analysis was conducted using the method recommended by the manufacturer. The results were normalized by the geometric mean of the expression of three reference genes, i.e., elongation factor 1-alpha (EF1α, X96555), 18 s ribosomal RNA (18 s, X52575) and beta-tubulin 3 (TUB, X54846). The relative expression of *PsPIPs* was calculated using the 2^−ΔΔCt^ method (Pfaffl 2001; Schmittgen and Livak 2008).

### Root and leaf hydraulic conductivity measurements

Root and leaf hydraulic conductivity (Lp_r_ and *K*_*leaf*_, respectively) was measured using the pressure chamber technique following Javot et al. (2003) and Postaire et al. (2010), with slight modifications. For Lp_r_ measurements, shoots were cut off below the first node of the plants, and the whole roots were bathed in nutrient solution in a pressure chamber (PMS, Corvallis, OR, USA). The hypocotyl was carefully threaded through the soft plastic washer of the metal lid. Pressure (P) that was generated by compressed air in steps of 0.1 MPa (up to 0.5 MPa) was slowly applied to the chamber, and the rate of exuded sap flow (*J*_v_) was determined. When *J*_v_ was plotted against the applied P, a linear relationship was observed for P values between 0.2 and 0.4 MPa (Fig. 4a). At the end of the measurement, the root system was removed and dry weight (DW) of the roots (after oven-dried at 70 °C for 72 h) was measured using a balance (FA2104N, Minqiao Instrument Co. Ltd, Shanghai, China). Lp_r_ (µL s^−1^ g^−1^ MPa^−1^) was calculated from the slope of the exuded sap flow rate versus pressure, divided by DW of the roots.

Similarly, for *K*_*leaf*_ determination, a detached mature compound leaf was inserted into a pressure chamber (PMS, Corvallis, OR, USA) filled with distilled water. The common petiole was carefully threaded through the soft plastic washer of the metal lid. Pressure was applied to the chamber in steps of 0.1 MPa (up to 0.5 MPa), using compressed air gas. This resulted in a flow of liquid (*J*_v_) entering through the leaf surface and exiting from the common petiole. When *J*_v_ was plotted against P, a linear relationship was observed for P values between 0.3 and 0.5 MPa (Fig. 5a). At the end of the measurement, leaves were scanned and the surface area (S) was measured using Image J software v1.42 (Bethesda, MD, USA). *K*_*leaf*_ (μL s^−1^ m^−2^ MPa^−1^) was calculated from the slope of the exuded sap flow rate versus pressure, divided by S of the leaves.

### Cell pressure probe measurements

Cell pressure probe (CPP) measurements were performed as described in Steudle (1993). In brief, pulled glass micro-capillary were beveled to a tip diameter of 5–7 µm, filled with silicone oil (type AS4; Wacker, Munich, Germany), and mounted to the CPP. To measure root cell hydraulic conductivity (Lp_rc_), root segment from plants grown in hydroponic condition was fixed by magnetic bars on a metal sledge which was covered with wet filter paper. An aerated nutrient solution was circulated along the root segment to maintain moisture. Root cells were punctured using a CPP, and cell sap entered the oil-filled micro-capillary forming a meniscus between cell sap and oil. Cell turgor pressure was restored by gently pushing the meniscus to a position close to the surface of the root, and the values of cell turgor pressure (P) were recorded by a computer (Ye et al. 2004). Half time of water exchange (T_1/2_) across cell membranes was obtained from hydrostatic pressure relaxation curves with the aid of the probe. After CPP measurements, average values of cell volume and surface area were obtained through microscopic analyses with root sections, assuming that cells had a cylindrical shape. Lp_rc_ was calculated according to the following equation:

1 $$ {\text{Lp}} = \frac{{V}}{{A}} \times \frac{\ln (2)}{{T_{1/2}^{w} (\varepsilon + \pi^{i} )}} $$

Here, V = cell volume; A = cell surface area; π^i^ = osmotic pressure of cell sap; ε = cell elastic modulus. π^i^ was calculated from the initial cell turgor (P_0_), as P_0_ = π^i^ − π^0^ (π^0^ = osmotic pressure of the medium measured with an osmometer); elastic modulus was determined from relative change of cell volume (ΔV/V) and the instantaneous change of cell turgor (ΔP):

2 $$ \varepsilon = V \times \frac{\Delta P}{\Delta V} $$

Where the change in cell volume (ΔV) was induced by moving the meniscus with the aid of the CPP, which was calculated from the length of meniscus movement in the micro-capillary using the eyepiece reticule of the microscope under a given magnification, and from the inner diameter of the capillary where the meniscus located (Steudle 1993).

For leaf cell hydraulic conductivity (Lp_lc_) measurements, a mature young leaf blade (still attached to the plant) was fixed onto a metal sledge. Leaf cells were punctured using a CPP, and water relation parameters such as T_1/2_, ε, and Lp_lc_ were determined as described above for root cell measurements.

### Statistical analysis

Results were presented as mean ± SD of three independent experiments. Statistical analyses were performed using SPSS 13.0 program (Chicago, IL, USA). Statistical significant differences were determined by t test at P < 0.05.

## Additional files

**Additional file 1: Table S1.** Sequences of degenerate oligo nucleotide primers designed from the known sequences of different plant PIP genes.

**Additional file 2: Table S2.** Sequences of gene-specific primers used for real-time RT-PCR amplification.

**Additional file 3: Figure S1.** Virus-induced gene silencing of *P. sativum* phytoene desaturase (*PsPDS*). (A) Leaves of a control plant inoculated with PEBV carrying a fragment of Bean yellow mosaic virus (pCAPE2-Con) remained green; (B) leaves of a plant inoculated with PEBV carrying a fragment of *PsPDS* (pCAPE2-PDS) showed a characteristic bleaching phenotype.

## Abbreviations

AQPs: aquaporins
CPP: cell pressure probe
*K*_*leaf*_: leaf hydraulic conductivity
Lp_lc_: leaf cell hydraulic conductivity
Lp_r_: root hydraulic conductivity
Lp_rc_: root cell hydraulic conductivity
PIP: plasma membrane intrinsic protein
q-RT-PCR: quantitative real-time PCR
T_1/2_: half-time of water exchange
VIGS: virus-induced gene silencing
