# Supplementary material for: Virus-induced plasma membrane aquaporin PsPIP2;1 silencing inhibits plant water transport of Pisum sativum
Source: Bot Stud. 2016 Aug 6;57:15. doi: 10.1186/s40529-016-0135-9 (PMC5430582; doi:10.1186/s40529-016-0135-9)
Supplement: Supplementary file 1 — Additional file 1: Table S1. Sequences of degenerate oligo nucleotide primers designed from the known sequences of different plant PIP genes. [file 40529_2016_135_MOESM1_ESM.docx]

**Table S1** Sequences of degenerate oligonucleotide primers designed from the known sequences of different plant PIP genes. Degenerate base S, M, R and Y represents C/G, A/C, A/G and C/T, respectively.

| Primer name Sequences of forward degenerate oligonucleotide primers |
| --- |
| *PsPIP1*  5´- ATGGAAGSMAARGAASAAGA -3´  *PsPIP2*  5´- ATGGCSAAAGAYGTTGA -3´ |
