# Supplementary material for: Virus-induced plasma membrane aquaporin PsPIP2;1 silencing inhibits plant water transport of Pisum sativum
Source: Bot Stud. 2016 Aug 6;57:15. doi: 10.1186/s40529-016-0135-9 (PMC5430582; doi:10.1186/s40529-016-0135-9)

**Figure S1** Virus-induced gene silencing of *P. sativum* phytoene desaturase (*PsPDS*). (A) Leaves of a control plant inoculated with PEBV (*Pea early browning virus*) carrying a fragment of Bean yellow mosaic virus (pCAPE2-Con) remained green; (B) leaves of a plant inoculated with PEBV carrying a fragment of *PsPDS* (pCAPE2-PDS) showed a characteristic bleaching phenotype.


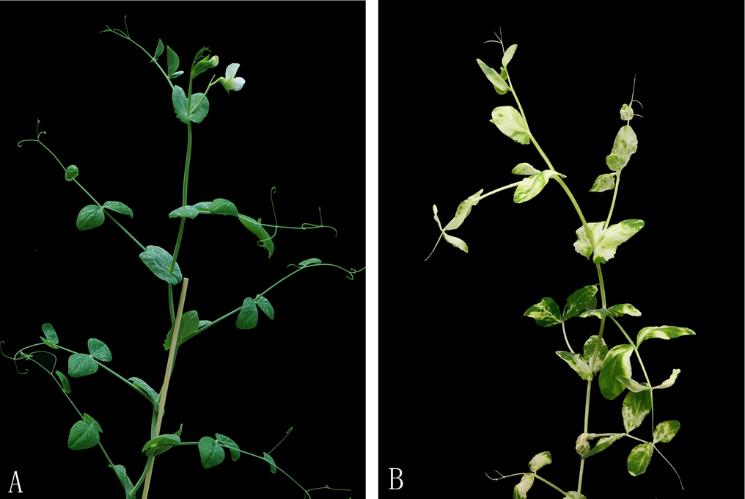

Supplement: Supplementary file 2 — Additional file 2: Table S2. Sequences of gene-specific primers used for real-time RT-PCR amplification. [file 40529_2016_135_MOESM2_ESM.docx]
