# Supplementary material for: Virus-induced plasma membrane aquaporin PsPIP2;1 silencing inhibits plant water transport of Pisum sativum
Source: Bot Stud. 2016 Aug 6;57:15. doi: 10.1186/s40529-016-0135-9 (PMC5430582; doi:10.1186/s40529-016-0135-9)
Supplement: Supplementary file 3 — Additional file 3: Figure S1. Virus-induced gene silencing of P. sativum phytoene desaturase (PsPDS). (A) Leaves of a control plant inoculated with PEBV carrying a fragment of Bean yellow mosaic virus (pCAPE2-Con) remained green; (B) leaves of a plant inoculated with PEBV carrying a fragment of PsPDS (pCAPE2-PDS) showed a characteristic bleaching phenotype. [file 40529_2016_135_MOESM3_ESM.docx]

**Table S2** Sequences of gene-specific primers used for real-time RT-PCR amplification.

| Primer name | Accession NO. | Sequences of forward and reverse primers | Amplicon length (bp) |
| --- | --- | --- | --- |
| *PsPIP1;1* | X54357 | 5´- AAACGTAGCGCCAGAGACTCTCATG-3´ | 192 |
|  |  | 5´- ATGGTCCAACCCAGAAAATCCGT-3´ |  |
| *PsPIP1;2* | KF770828 | 5´- TTACTGGAACTGGTATCAACCCTGCT-3´ | 186 |
|  |  | 5´- CCGTTGGATCATATCAACTTGACTTG-3´ |  |
| *PsPIP2;1* | AJ243307 | 5´- CGCTGTGATCTTAAACCAGGGGA-3´ | 214 |
|  |  | 5´AATTAACACCATATCTTCCTTGGCAAA-3´ |  |
| *PsPIP2;2* | KF770829 | 5´- GATCCTTCAGGAGCAATGCTTAATG-3´ | 218 |
|  |  | 5´- TGCTTATCTACAGTTTTGAGGACCCC-3´ |  |
| *PsPIP2;3* | KF770830 | 5´- TTGGCAACCATCCCAGTCACC-3´ | 188 |
|  |  | 5´- TCACTTCATCTTCTTTCCATTAGCACTC -3´ |  |
| *TUB* | X54846 | 5´- TTGCGACGAGCACGGCATAGA-3´ | 215 |
|  |  | 5´- CGGACTGACCGAAGACGAAGTTATC-3´ |  |
| *EF* | X96555 | 5´- CAGGCTGATTGTGCTGTCCTTATTAT-3´ | 191 |
|  |  | 5´- ACGATTTCCTCATACCTGCCCTT-3´ |  |
| *18S rRNA* | X52575 | 5´- CTCTGCCTGTTGCTTTGATGATTC-3´ | 194 |
